# Supplementary material for: Analysis and Prediction of Electrospun Nanofiber Diameter Based on Artificial Neural Network
Source: Polymers (Basel). 2023 Jun 25;15(13):2813. doi: 10.3390/polym15132813 (PMC10346665; doi:10.3390/polym15132813)
Supplement: Supplementary file 1 [file polymers-15-02813-s001.zip › polymers-2431437-supplementary.pdf]

Supporting Information

Analysis and Prediction of Electrospun Nanofiber Diameter Based on Artificial Neural Network

Ming Ma [1,2], Huchen Zhou [2,3], Suhan Gao [2,3], Nan Li [2,4,*], Wenjuan Guo [2,5] and Zhao Dai [2,3,*]

[1] School of Life Sciences, Tiangong University, Tianjin 300387, China; maming@tiangong.edu.cn
[2] State Key Laboratory of Separation Membranes and Membrane Processes, Tiangong University, Tianjin 300387, China
[3] School of Chemical Engineering and Technology, Tiangong University, Tianjin 300387, China
[4] School of Chemistry, Tiangong University, Tianjin 300387, China
[5] School of Pharmaceutical Sciences, Tiangong University, Tianjin 300387, China
* Correspondence: linan@tiangong.edu.cn (N.L.); daizhao@tiangong.edu.cn (Z.D.)

Table S1 Electrospinning parameters of PAN and measured fiber diameter

Exp. No.	Concentration (wt%)	Voltage (kV)	receiving distance (cm)	Injection speed (mL/h)	Nanofiber diameter (nm)
1	6	10	18	0.4	125.13
2	6	12	18	0.4	119.46
3	6	14	18	0.4	128.53
4	6	16	18	0.4	106.69
5	6	18	12	0.4	147.72
6	6	18	14	0.4	138.81
7	6	18	16	0.4	142.41
8	6	18	18	0.4	150.67
9	6	18	18	0.1	118.18
10	6	18	18	0.2	118.31
11	6	18	18	0.3	118.05
12	6	18	18	0.5	116.27
13	6	18	20	0.4	123.67
14	6	18	22	0.4	122.26
15	6	20	18	0.4	133.62
16	6	22	18	0.4	108.47
17	6	22	20	0.5	95.71
18	8	10	18	0.4	131.12
19	8	10	20	0.4	162.59
20	8	12	18	0.4	139.7
21	8	12	20	0.4	168.37
22	8	14	18	0.4	140.91
23	8	14	20	0.4	153.01
24	8	16	18	0.4	155.35
25	8	16	20	0.4	179.42
26	8	18	18	0.4	148.36
27	8	18	20	0.4	171.55
28	8	18	20	0.2	105.46
29	8	20	12	0.4	156.83
30	8	20	12	0.5	105.73
31	8	20	14	0.4	181.29

Exp. No.	Concentration (wt%)	Voltage (kV)	receiving distance (cm)	Injection speed (mL/h)	Nanofiber diameter (nm)
32	8	20	16	0.4	147.11
33	8	20	18	0.4	144.55
34	8	20	18	0.1	144.91
35	8	20	18	0.2	197.68
36	8	20	18	0.3	189.62
37	8	20	18	0.5	144.52
38	8	20	20	0.4	188.43
39	8	20	22	0.4	158.91
40	8	22	18	0.4	181.41
41	8	22	18	0.5	174.32
42	8	22	20	0.4	168.94
43	8	22	22	0.4	166.68
44	10	10	18	0.4	148.38
45	10	12	18	0.4	177.68
46	10	14	18	0.4	180.47
47	10	16	18	0.4	223.4
48	10	18	12	0.1	295.41
49	10	18	12	0.2	298.8
50	10	18	12	0.3	301.08
51	10	18	12	0.4	279.47
52	10	18	12	0.5	236.48
53	10	18	14	0.1	260.33
54	10	18	14	0.2	258.89
55	10	18	14	0.3	144.95
56	10	18	14	0.4	219.29
57	10	18	14	0.5	190.31
58	10	18	16	0.1	207.28
59	10	18	16	0.2	199.66
60	10	18	16	0.3	188.14
61	10	18	16	0.4	285.5
62	10	18	16	0.5	274.51
63	10	18	18	0.1	306.36

Exp. No.	Concentration (wt%)	Voltage (kV)	receiving distance (cm)	Injection speed (mL/h)	Nanofiber diameter (nm)
64	10	18	18	0.2	329
65	10	18	18	0.3	313.54
66	10	18	18	0.4	313.46
67	10	18	18	0.5	199.07
68	10	18	20	0.1	287.63
69	10	18	20	0.2	291.42
70	10	18	20	0.3	323.13
71	10	18	20	0.4	307.75
72	10	18	20	0.5	265.06
73	10	18	22	0.1	239.69
74	10	18	22	0.2	217.2
75	10	18	22	0.3	243.3
76	10	18	22	0.4	220.68
77	10	18	22	0.5	187.52
78	10	20	18	0.1	237.51
79	10	20	18	0.2	262.33
80	10	20	18	0.3	256.41
81	10	20	18	0.4	203.44
82	10	22	12	0.3	203.89
83	10	22	18	0.4	207.67
84	10	22	18	0.5	209.02
85	12	10	20	0.4	282.14
86	12	12	20	0.4	291.99
87	12	14	20	0.4	276.03
88	12	16	20	0.4	271.59
89	12	18	12	0.4	237.01
90	12	18	14	0.4	348.77
91	12	18	16	0.4	342.9
92	12	18	18	0.1	306.36
93	12	18	18	0.2	267.6
94	12	18	18	0.3	276.43
95	12	18	18	0.4	291.45

Exp. No.	Concentration (wt%)	Voltage (kV)	receiving distance (cm)	Injection speed (mL/h)	Nanofiber diameter (nm)
96	12	18	18	0.5	312.45
97	12	18	20	0.4	294.61
98	12	18	22	0.4	290.63
99	12	20	20	0.4	308.12
100	12	20	20	0.3	246.53
101	12	22	18	0.2	256.58
102	12	22	20	0.4	325.36
103	14	10	18	0.4	309.72
104	14	12	18	0.4	453.94
105	14	14	18	0.4	371.6
106	14	16	18	0.4	362.43
107	14	18	12	0.4	389.78
108	14	18	14	0.4	357.36
109	14	18	16	0.4	338.89
110	14	18	18	0.4	365.83
111	14	18	18	0.1	385.95
112	14	18	18	0.2	362.9
113	14	18	18	0.3	396.14
114	14	18	18	0.5	404.59
115	14	18	20	0.4	360.89
116	14	18	22	0.4	349.76
117	14	20	18	0.4	367.47
118	14	22	18	0.4	382.01
119	14	22	20	0.4	402.29
120	16	10	20	0.4	684.39
121	16	12	20	0.4	860
122	16	14	20	0.4	522.22
123	16	16	20	0.4	485.78
124	16	18	12	0.4	464.69
125	16	18	14	0.4	489.3
126	16	18	16	0.4	471.99
127	16	18	18	0.5	436.32

Exp. No.	Concentration (wt%)	Voltage (kV)	receiving distance (cm)	Injection speed (mL/h)	Nanofiber diameter (nm)
128	16	18	18	0.4	455.18
129	16	18	18	0.3	458.58
130	16	18	18	0.2	539.3
131	16	18	18	0.1	627.54
132	16	18	20	0.4	473.28
133	16	18	22	0.4	522.58
134	16	20	20	0.4	456.13
135	16	22	20	0.5	437.39
136	16	22	20	0.4	521.84
137	16	22	22	0.5	403.67
